# Quercetin Increases Mitochondrial Biogenesis and Reduces Free Radicals in Neuronal SH-SY5Y Cells

**DOI:** 10.3390/nu14163310

**Published:** 2022-08-12

**Authors:** Chia-Ling Ho, Ning-Jo Kao, Ching-I Lin, Tzu-Wen L. Cross, Shyh-Hsiang Lin

**Affiliations:** 1 School of Nutrition and Health Sciences, Taipei Medical University, Taipei 110, Taiwan; 2 Department of Nutrition and Health Sciences, Kainan University, Taoyuan 338, Taiwan; 3 Department of Nutrition Science, Purdue University, West Lafayette, IN 47907, USA; 4 Master Program in Food Safety, Taipei Medical University, Taipei 110, Taiwan

**Keywords:** Aβ, oxidative stress, quercetin, mitochondrial biogenesis, neurodegenerative disease

## Abstract

Alzheimer’s disease (AD) is a common neurodegenerative disorder that causes dementia and affects millions of people worldwide. The mechanism underlying AD is unclear; however, oxidative stress and mitochondrial biogenesis have been reported to be involved in AD progression. Previous research has also reported the reduction in mitochondrial biogenesis in the brains of patients with AD. Quercetin (QE), a type of polyphenol, has been found to be capable of increasing mitochondrial biogenesis in the body. Accordingly, we explored whether QE could reduce amyloid beta (Aβ) accumulation caused by hydrogen peroxide (H_2_O_2_)-induced oxidative stress in SH-SY5Y cells. Our results revealed that QE stimulated the expression of mitochondrial-related proteins such as SIRT1, PGC-1α, and TFAM and subsequently activated mitochondrial biogenesis. Additionally, QE increased ADAM10 expression but reduced H_2_O_2_-induced reactive oxygen species production, apoptosis, β-site amyloid precursor protein cleaving enzyme 1 expression, and Aβ accumulation in the SH-SY5Y cells. These findings indicate that QE can effectively elevate mitochondrial biogenesis-related proteins and reduce the damage caused by oxidative stress, making it a promising option for protecting neuronal cells.

## 1. Introduction

Aging is characterized by the progressive loss of tissue and organ function, which is related to the accumulation of reactive oxygen species (ROS)-mediated oxidative damage [1,2]. Oxidative stress not only affects aging but is also involved in several age-related conditions such as cardiovascular disease, chronic obstructive pulmonary disease, chronic kidney disease, cancer, and neurodegenerative diseases. Alzheimer’s disease (AD) is a common neurodegenerative disease that causes dementia and affects millions of people worldwide; currently, only symptomatic treatments are available for AD [3]. Although the mechanism underlying AD is unclear, oxidative stress and mitochondrial biogenesis have been reported to be involved in the progression of AD [1,4]. Oxidative stress is characterized by the overproduction of ROS, which can induce mitochondrial DNA (mtDNA) mutations, damage the mitochondrial respiratory chain, alter membrane permeability, and influence Ca^2+^ homeostasis and mitochondrial defense systems. Hydrogen peroxide (H_2_O_2_), a product of superoxide dismutase (SOD) dismutation, is among the ROS that engender oxidative stress [5]. H_2_O_2_ accumulation may cause protein oxidization, cell membrane lipid oxidation, and DNA strand breakage [6], which may lead to cell apoptosis [7].

Mitochondria play a key role in the pathogenesis of many diseases [8,9,10,11,12] and in aging [13]. Studies have reported an age-related decrease in the number of mitochondria in the brain by using electron microscopy [14], enzymatic methods [15,16,17], and mtDNA-encoded gene expression estimation methods [18,19]. In addition to the number of mitochondria, the functional state of mitochondria—which depends on mitochondrial biogenesis and dynamics, including fission, fusion, and mitophagy—plays a major role in disease pathogenesis. The dysregulation of mitochondrial biogenesis and dynamics leads to an age-related decrease in mitochondrial volume density and oxidative capacity per mitochondrial volume [20]. The coordination between these processes is controlled by several mutually regulated signaling cascades, including the nuclear respiratory factor 1 (NRF1)/nuclear respiratory factor 2 (NRF2)/antioxidant response element (ARE)/mitochondrial transcription factor A (TFAM) cascade [21].

NRF1, NRF2, and TFAM are regulated by peroxisome proliferator-activated receptor gamma coactivator 1-alpha (PGC-1α). These factors mutually regulate mitochondrial biogenesis [22,23,24,25,26]. A study observed reduced PGC-1α and TFAM levels in the brains of patients with AD [27]. PGC-1α and sirtuin 1 (SIRT1) were reported to play crucial roles in mitochondrial function, energy regulation, and metabolic homeostasis [28]. Another study revealed that the blockade of mitochondrial function and energy production in the brain may increase the levels of amyloid beta (Aβ)-related enzymes such as β-site amyloid precursor protein (APP) cleaving enzyme 1 (BACE1), resulting in the accumulation of Aβ, one of the two major pathologic features of AD [29].

Quercetin (QE) is a natural flavonoid. It possesses antioxidant properties [30,31] and has been found to increase mitochondrial biosynthesis [32] and reduce cell apoptosis [33]. A study demonstrated that mice that consumed QE-containing diets exhibited increased mtDNA copy numbers [32]. This study was conducted to explore possible strategies for reducing oxidative damage in the brain in order to treat neurodegeneration. SH-SY5Y cells are considered the most representative cellular model for investigations into AD [34,35,36,37,38,39,40]. SH-SY5Y cells were also used to characterize the main pathologic components of neurofibrillary tangles. Neurofibrillary tangles are paired helical filaments of the highly phosphorylated microtubule- associated protein tau [41,42,43]. Accordingly, we investigated the mechanism through which QE reduces Aβ accumulation in H_2_O_2_-treated SH-SY5Y cells.

## 2. Materials and Methods

### 2.1. Cell Culture

This study used the SH-SY5Y human neuroblastoma cell line (American Type Culture Collection, Manassas, VA, USA), a well-established model for studying neurodegenerative diseases. The SH-SY5Y cells were cultured in Dulbecco’s modified Eagle’s medium (DMEM; Life Technologies, Grand Island, NY, USA) that was supplemented with F12 (Life Technologies, NY, USA) containing 10% fetal bovine serum (FBS; Biowest, Nuaillé, France), 1% glutamine (Bionovas, Toronto, ON, Canada), and supplemented with 0.01% penicillin–streptomycin (Sigma, St. Louis, MO, USA) at 37 °C in a 5% CO_2_ incubator. The medium was replaced every 2~3 days. Each aliquot (vial) of cells was grown for no more than 10 passages.

### 2.2. Sirtinol, QE, and H_2_O_2_ Treatment

Experiments were performed at 80% cell confluence. Then cells were incubated with QE at final concentrations of 0, 2.5, 5, 7.5, and 10 μM according to the anti-inflammation effect in vivo [44] for 24 h; followed by washing and incubation with 40 μM H_2_O_2_ for another 24 h. Additionally, sirtinol (Sigma, St. Louis, MO, USA), an inhibitor of the SIRT1-signaling pathway, was used in some of the experiments to elucidate the role of SIRT1 on QE-stimulated effects. Fifty μM of sirtinol (Sigma, St. Louis, MO, USA) was added to the medium 1 h prior to exposure to the QE treatment. In this study, we used 99% ethanol for dissolving quercetin (Quercetin Dihydrate, Wako, Japan). The final concentration of the stock solution was 20 mM. A concentration of 0 μM quercetin was accomplished by adding 99% ethanol (Sigma, St. Louis, MO, USA). The final concentration of 99% ethanol was diluted under 0.05% in all cell cultures.

### 2.3. Cell Viability Assay

Cell viability was assessed using the trypan blue exclusion assay. Briefly, 50 μL of 0.4% trypan blue solution was added to 50 μL of cell suspension in a culture medium. The suspension was gently mixed and placed in a hemacytometer for cell counting. Viable and dead cells were identified and counted under a light microscope. Blue cells failing to exclude dyes were considered nonviable and transparent cells were considered viable. The percentage of viable cells was calculated according to the total number of cells (viable plus nonviable)

### 2.4. Measurement of Mitochondrial Biogenesis

The SH-SY5Y cells were plated in a six-well plate at a density of 8 × 10^5^ cells. To determine the number of mitochondria, a suspension of cells in growth medium was loaded with 100 μM MitoTracker Red FM (Phycoerythrin, PE red) for 45 min at 37 °C. Fluorescence intensity was measured at an excitation wavelength of 581 nm and emission wavelength of 644 nm by using a flow cytometer (BD FACSCanto II system, BD Biosciences, San Jose, CA, USA). The number of mitochondria was determined by comparing the intensity of the fluorescence signals produced by 1 × 10^4^ cells.

### 2.5. Measurement of Cellular Adenosine Triphosphate Production

Adenosine triphosphate (ATP) was measured using the ATP Determination Kit (A22066, Life Technologies, NY, USA). Briefly, the cells (1 × 10^6^ cells/mL) were resuspended in reaction buffer containing 1 mM dithiothreitol, 0.5 mM luciferin, and 12.5 μg/mL luciferase. The suspension was mixed gently, after which the corresponding readings were taken using a luminometer (Turner Designs, Sunnyvale, CA, USA). ATP concentrations were calculated using an ATP standard curve. Cellular ATP levels were quantified relative to protein concentration.

### 2.6. Measurement of Intracellular ROS

Intracellular ROS were assayed using 2′,7′-dichlorofluorescin diacetate (DCFH-DA). After termination of the treatments, the cells were harvested using 1× trypsin solution before being rinsed twice in phosphate-buffered saline (PBS). Cell pellets were resuspended in 0.5 mL of PBS containing 15 μM DCFH-DA and incubated at 37 °C for 45 min. The intracellular ROS levels were measured using a flow cytometer (BD FACSCanto II system).

### 2.7. Measurement of Cellular Apoptosis

Apoptotic and necrotic cells were quantified using annexin V binding and propidium iodide (PI) uptake in accordance with the manufacturer’s instructions. After incubation, the cells were collected using centrifugation and washed twice with 5 mL of cold 1× PBS. The cells were resuspended in 1× binding buffer at a concentration of 1 × 10^6^ cells/mL. Approximately 0.5 mL of each sample was transferred to a 5 mL Falcon tube. Subsequently, 1× binding buffer (0.1 mL) was added to each tube. Annexin V FITC and PI were added to each sample and incubated in the dark for 15 min. The apoptotic level was analyzed using the flow cytometer (BD FACSCanto II system).

### 2.8. Western Blotting

Cell lysates were prepared in 1× RIPA Buffer, pH7.6 (150 mM NaCl, 1.00% Triton X-100, 0.50% Sodium deoxycholate, 0.10% SDS, 50 mM Tris-HCl) containing protease inhibitors. The protein concentration was estimated with a BCA Protein Assay Kit (Milpitas, CA, USA) using bovine serum albumin as the standard. Equal quantities (30 μg) of protein were electrophoresed on 10–15% gradient polyacrylamide gels and transferred onto Immobilon-P membranes (Millipore, Bedford, MA, USA). The membranes were blocked with 5% nonfat milk and subsequently incubated in mouse anti-SIRT1 (1:8000, Abcam, Cambridge, MA, USA), rabbit anti-PGC-1α (1:1000, Abcam, Cambridge, MA, USA), rabbit anti-TFAM (1:1000, Cell Signaling Technology, Danvers, MA, USA), rabbit anti-APP (1:1000, Merck Millipore, Darmstadt, Germany), rabbit anti-ADAM10 (1:1000, Merck Millipore, Darmstadt, Germany), rabbit anti-BACE (1:1000, Merck Millipore, Darmstadt, Germany), rabbit anti-Aβ (1:1000, Merck Millipore, Darmstadt, Germany), rabbit anti-active-caspase-3 (Abcam, Cambridge, MA, USA), and mouse antibeta actin (1:5000, Sigma, St. Louis, MO, USA) overnight at 4 °C. Immunoreactivity was determined through probing with horseradish peroxidase-conjugated antimouse (1:80,000, Sigma, St. Louis, MO, USA) or antirabbit immunoglobulin G (1:5000, Sigma, St. Louis, MO, USA), followed by exposure to enhanced chemiluminescence detection reagents (Merck KGaA, Darmstadt, Germany).

### 2.9. Statistical Analysis

Statistical analyses were performed using a one-way analysis of variance and an independent *t*-test, followed by Duncan’s post hoc test. A *p*-value of <0.05 was considered statistically significant.

## 3. Results

### 3.1. Effects of QE on Cellular ROS Production, Mitochondrial Biogenesis, and ATP Production in SH-SY5Y Cells with H_2_O_2_-Induced Oxidative Stress

**Figure 1:**
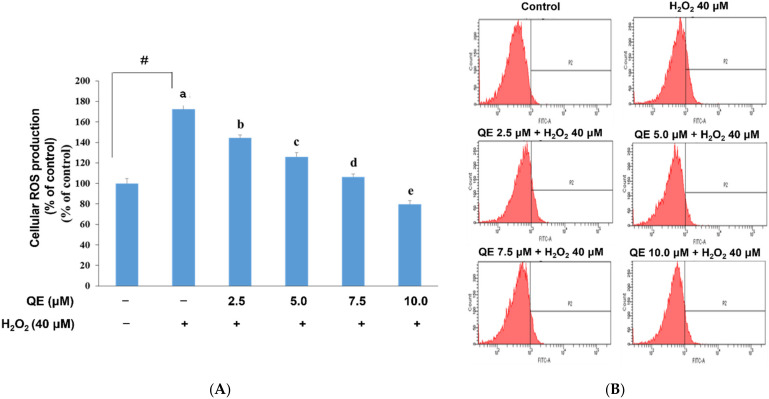
Effects of quercetin on the cellular ROS production following H_2_O_2_-induced oxidative stress in the SH-SY5Y cells. (**A**) Quantitative results of the ROS production in each group according to (**B**). Representative profiles of the ROS production detected by flow cytometry using DCF-DA assay. The SH-SY5Y cells were incubated with 40 μM H_2_O_2_ for 24 h after the addition of 0, 2.5, 5.0, 7.5, and 10.0 μM quercetin for 24 h. Data were presented as mean ± SD. # Data are analyzed using an independent *t*-test and are significantly different from the control group. Bars of 0, 2.5, 5.0, 7.5, and 10.0 μM quercetin with different letters significantly differ. Statistical analysis was performed using a one-way ANOVA, followed by a least significant difference post hoc test (*n* = 3).

Mitochondria are considered to be particularly susceptible to ROS attack associated with oxidative stress [45]. Persistent mtDNA damage ultimately leads to mutations in the mitochondrial genome [45] and engenders further mitochondrial dysfunction, which induces and aggravates diseases. In recent decades, QE has been found to possess an ROS reduction ability [46]. To clearly understand the relationship of ROS production, mitochondrial biogenesis, and neuroprotective mechanisms of QE, we examined cellular ROS and mitochondrial biogenesis in SH-SY5Y cells with H_2_O_2_-induced oxidative stress, which were precultured with QE and examined using DCF-DA and the phycoerythrin red assay separately. According to our preliminary experiment, in which the cells were treated with 25, 30, 35, 40, and 45 μM H_2_O_2_, the results (figures not shown) showed that the survival rates of the cells were 69.3 ± 6.8%, 66.9 ± 2.3%, 67.1 ± 7.5%, 53.4 ± 5.2%, and 37.8 ± 1.7%. Furthermore, the rate of cell apoptosis as shown in the expression of caspase-3 was elevated under 40 μM of H_2_O_2_ treatment. Thus, we chose 40 μM of H_2_O_2_ as our treatment. We observed that the ROS production level in cells that were treated with 40 μM H_2_O_2_ without being precultured with QE was significantly higher than that in the control group. In contrast, H_2_O_2_-induced ROS expression was significantly reduced in cells precultured with QE at 2.5, 5.0, 7.5, and 10.0 μM separately for 24 h (Figure 1A,B). According to the previous study [44], QE showed an in vivo effect of anti-inflammation at a concentration of 12.9 ± 1.3 μM in the blood of transgenic mice. Therefore, our study mimics the physical conditions of those mice, we chose 10 μM of quercetin as a high concentration to determine the results in an in vitro experiment.

**Figure 2:**
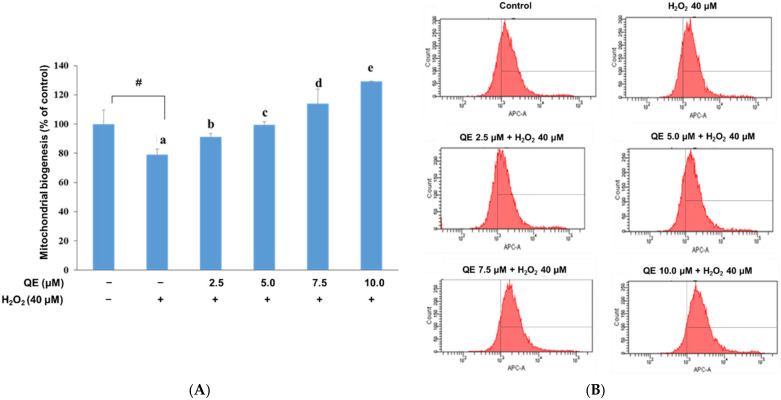
Effects of quercetin on mitochondrial biogenesis following H_2_O_2_-induced oxidative stress in the SH-SY5Y cells. (**A**) Quantitative results of mitochondrial biogenesis in each group according to (**B**). (**B**) Representative profiles of the mitochondrial biogenesis detected by flow cytometry using Mito Tracker Red FM. The SH-SY5Y cells were incubated with 40 μM H_2_O_2_ for 24 h after the addition of 2.5, 5.0, 7.5, and 10.0 μM quercetin for 24 h. Data were presented as mean ± SD. # Data are analyzed using an independent *t*-test and are significantly different from the control group. Bars of 0, 2.5, 5.0, 7.5, and 10.0 μM quercetin with different letters significantly differ. Statistical analysis was performed using a one-way ANOVA, followed by a least significant difference post hoc test (*n* = 3).

**Figure 3:**
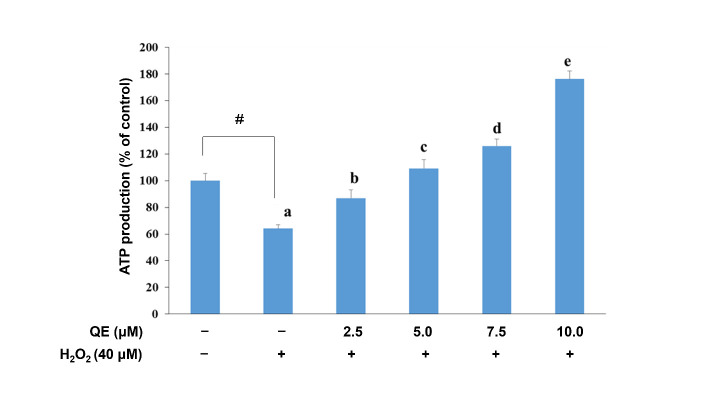
Effects of quercetin and H_2_O_2_ on cellular ATP production. Data were presented as mean ± SD. # Data are analyzed using an independent *t*-test and are significantly different from the control group. Bars of 0, 2.5, 5.0, 7.5, and 10.0 μM quercetin with different letters significantly differ. Statistical analysis was performed using a one-way ANOVA, followed by a least significant difference post hoc test (*n* = 3).

Cellular ROS overexpression was previously demonstrated to be associated with a decrease in mitochondrial biogenesis [45]. QE significantly raised mitochondrial biogenesis in the H_2_O_2_-induced SH-SY5Y cells in a dose-dependent manner (Figure 2A,B). Mitochondrial function is essential for ATP supply, especially in response to different cellular stressors. Impaired mitochondrial function is associated with alterations in ATP production, cellular synthetic and secretory function, cellular redox homeostasis, and nuclear gene expression [47,48]. We explored whether QE could increase mitochondrial biogenesis; therefore, we needed to examine whether the QE effect would be attenuated by the H_2_O_2_-induced oxidative stress in the SH-SY5Y cells. We observed that ATP production was significantly reduced in the cells with H_2_O_2_-induced oxidative stress (Figure 3). Comparatively, ATP production was significantly increased in the cells that were precultured with QE at 2.5, 5.0, 7.5, and 10.0 μM for 24 h and then incubated with 40 μM H_2_O_2_ for 24 h.

### 3.2. Effects of QE on Mitochondrial Biogenesis-Related Protein Expression in SH-SY5Y Cells with H_2_O_2_-Induced Oxidative Stress

The loss of mitochondrial biogenesis was previously considered to play a major role in AD progression [48]. This process is related to PGC-1α modulation. PGC-1α is a fasting-induced transcriptional coactivator recruited during peroxisome proliferator-activated receptor stimulation [49]. PGC-1α deacetylation caused by SIRT1 results in the upregulation of its transcriptional function [50,51,52]. SIRT1, an oxidized nicotinamide adenine dinucleotide (NAD^+^)-dependent histone deacetylase, is essential for maintaining cellular survival, cognitive function, and synaptic plasticity [53]. In particular, SIRT1 regulates diverse processes such as apoptosis, cellular senescence, glucose homeostasis, and aging in oxidative stress environments [54].

**Figure 4:**
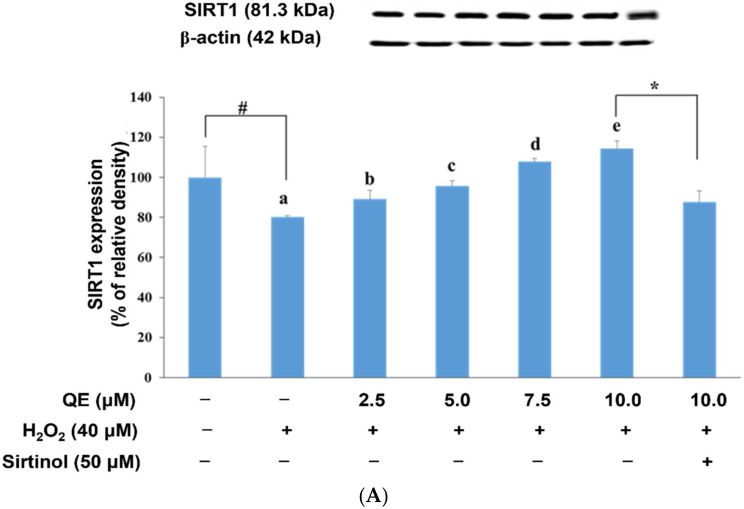

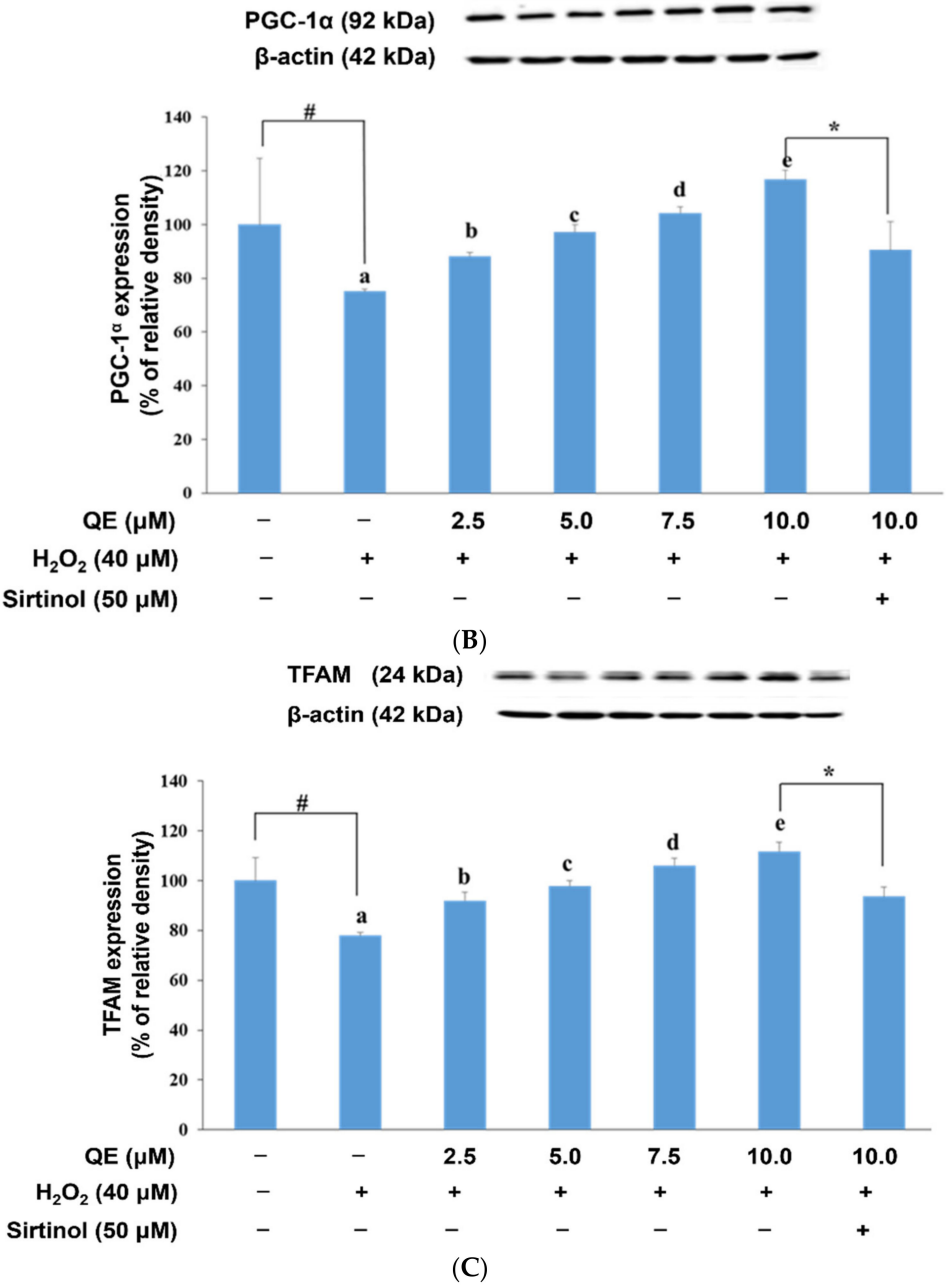
Effects of quercetin and H_2_O_2_ on SIRT1 (**A**), PGC-1α (**B**) and TFAM (**C**) protein expression. Data were presented as mean ± SD. # Data are analyzed using an independent *t*-test and are significantly different from the control group. * Data are analyzed using an independent *t*-test and are significantly different from the group added 10 μM quercetin but without sirtinol. Bars of 0, 2.5, 5.0, 7.5, and 10.0 μM quercetin with different letters significantly differ. Statistical analysis was performed using a one-way ANOVA, followed by a least significant difference post hoc test (*n* = 3).

We used Western blotting to determine the expression of proteins related to mitochondrial biogenesis in SH-SY5Y cells precultured with QE and subjected to H_2_O_2_-induced oxidative stress. Specifically, the SH-SY5Y cells were precultured with 2.5, 5.0, 7.5, and 10.0 μM QE separately for 24 h, followed by exposure to 40.0 μM H_2_O_2_ for 24 h. The results revealed that the expression of mitochondrial biogenesis-related proteins such as SIRT1, PGC-1α, and TFAM in the cells decreased significantly (*p* < 0.05) due to H_2_O_2_ treatment. The addition of QE elevated SIRT1, PGC-1α, and TFAM expression significantly in a dose-dependent manner in the cells treated with H_2_O_2_ as shown in Figure 4A–C.

### 3.3. QE Reduced the Expression of Proteins Involved in the APP Amyloidogenic Pathway in SH-SY5Y Cells with H_2_O_2_-Induced Oxidative Stress

Both APP and Aβ are located in the mitochondria [27,55,56]. Aβ not only causes significant oxidative damage to mtDNA but also leads to impaired mtDNA gene expression [57]. Aβ is generated from APP through a sequential proteolytic process initiated by BACE1 or β-secretase. BACE1 cleavage of APP is the rate-limiting step in Aβ production. In addition, BACE could modulate ADMA-10, another main enzyme involved in Aβ synthesis. Increased BACE expression could promote Aβ production. However, ADMA-10 overexpression could reduce Aβ generation.

**Figure 5:**
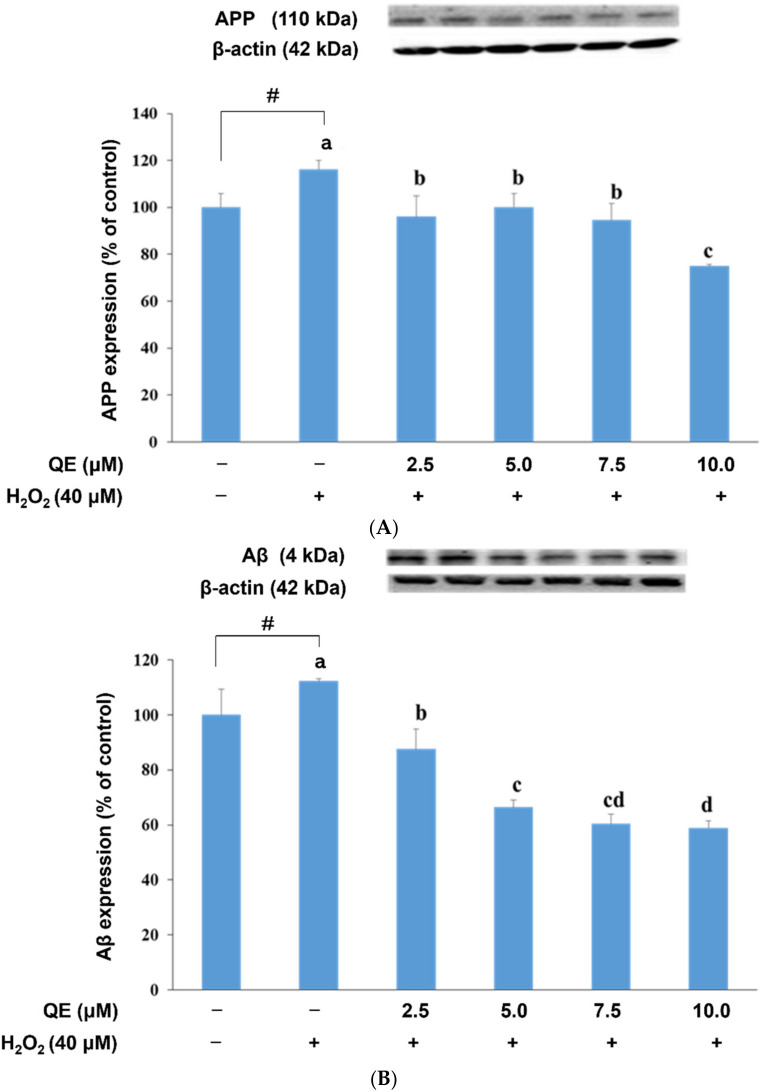

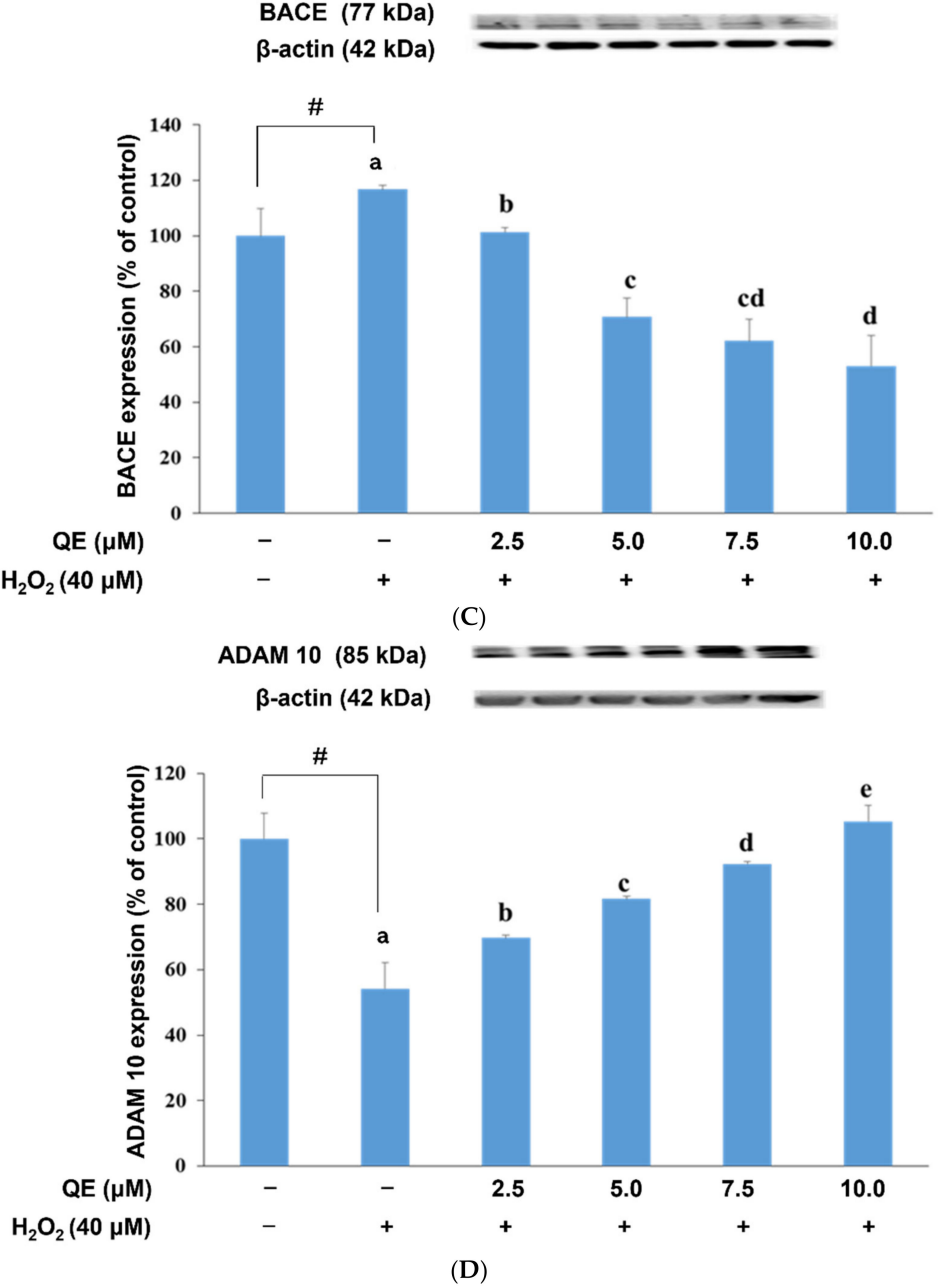
Effects of quercetin and H_2_O_2_ on APP (**A**), Aβ (**B**), BACE-1 (**C**) and ADMA (**D**) protein expression. Data were presented as mean ± SD. # Data are analyzed using an independent *t*-test and are significantly different from the control group. Bars of 0, 2.5, 5.0, 7.5, and 10.0 μM quercetin with different letters significantly differ. Statistical analysis was performed using a one-way ANOVA, followed by a least significant difference post hoc test (*n* = 3).

We examined the influence of QE on APP, Aβ, BACE, and ADMA-10 expression in the SH-SY5Y cells with H_2_O_2_-induced oxidative stress (Figure 5). APP expression was significantly increased in these cells; however, QE reduced the APP expression levels (Figure 5A). Similar results were observed for Aβ expression, signifying that QE might reduce APP expression and subsequently engender the reduction in Aβ expression (Figure 5B). QE also reduced BACE expression significantly (Figure 5C) but increased ADMA-10 expression (Figure 5D).

### 3.4. QE Reduced Cellular Apoptosis and Active Caspase-3 Expression in SH-SY5Y Cells with H_2_O_2_-Induced Oxidative Stress

**Figure 6:**
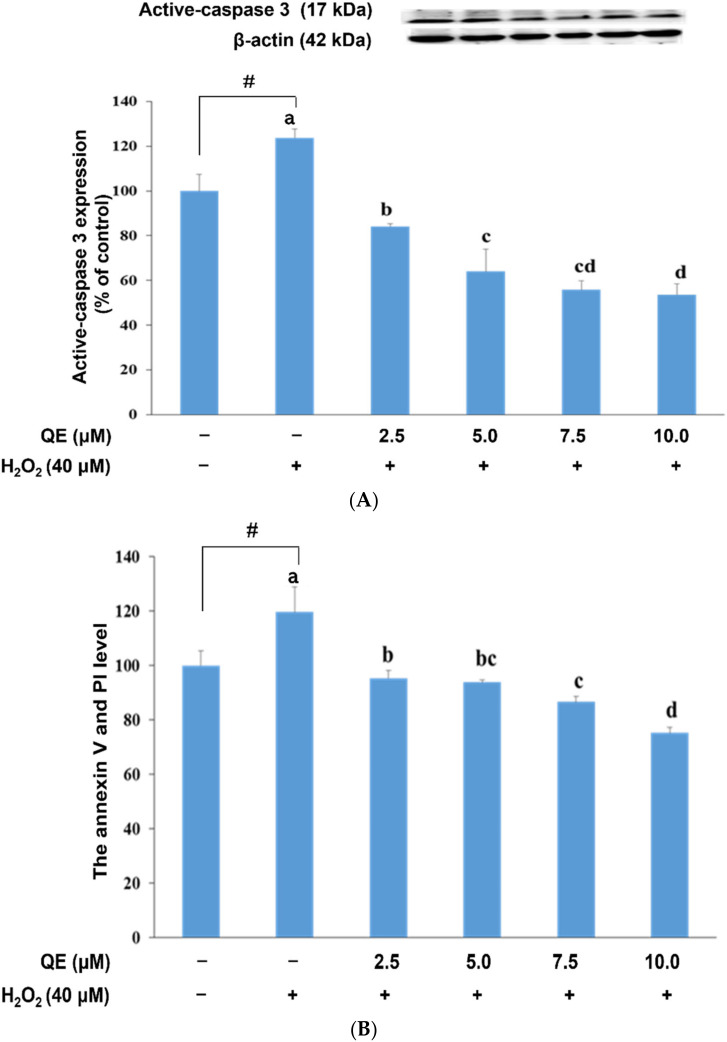

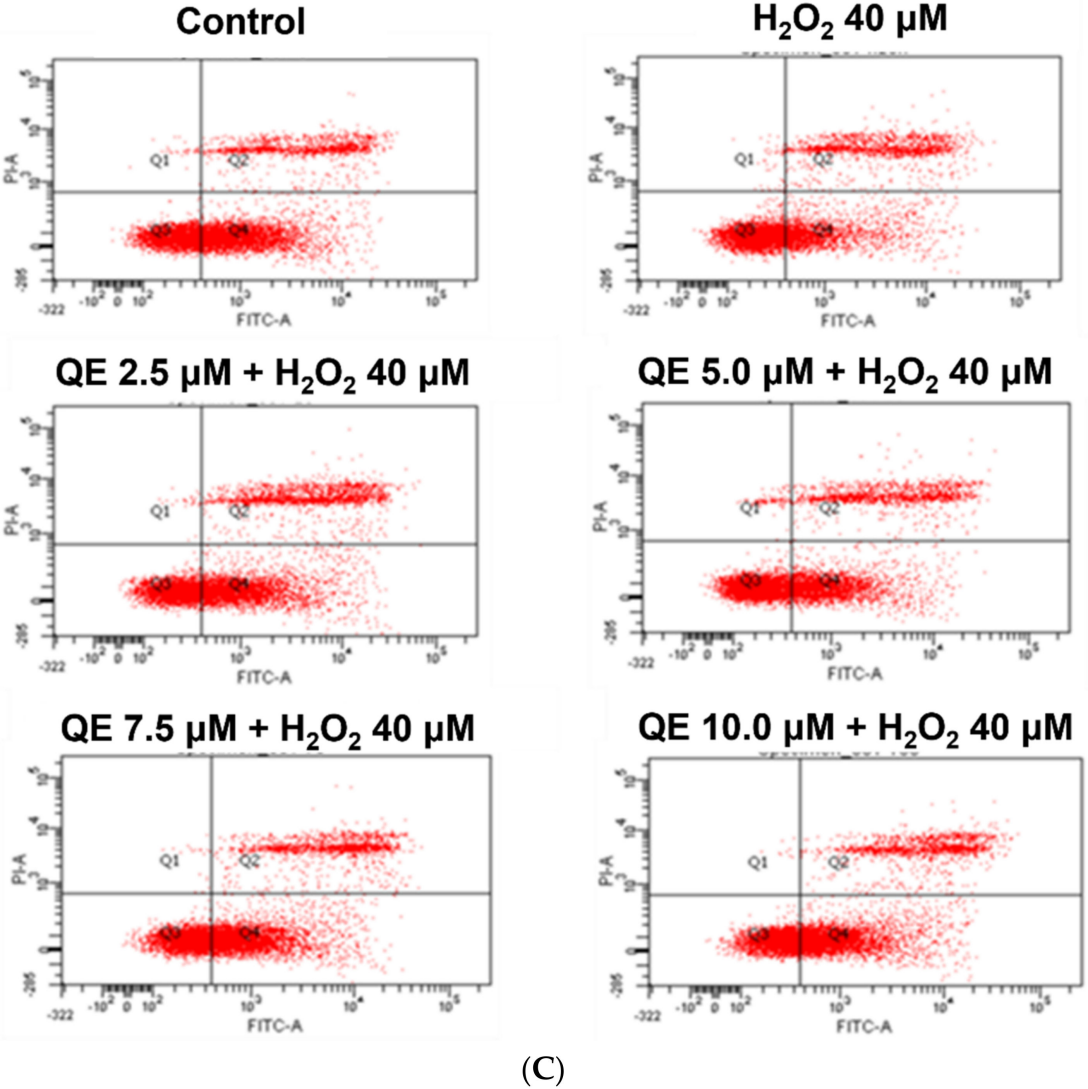
Effects of quercetin on H_2_O_2_-induced changes in cell apoptosis. (**A**) Western blot analysis of active-caspase 3 expression in SH-SY5Y cells. (**B**) Percentages of apoptotic cells in each group quantified from (**C**). (**C**) Representative profiles of cell apoptosis detected by flow cytometry with Annexin V/propidium iodide double-staining. The cells were cultured with 0.0, 2.5, 5.0, 7.5 and 10.0 μM of quercetin for 24 h and then treated with 40 μM of H_2_O_2_ for 24 h. Data were presented as mean ± SD. # Data are analyzed using an independent *t*-test and are significantly different from the control group. Bars of 0, 2.5, 5.0, 7.5, and 10.0 μM quercetin with different letters significantly differ. Statistical analysis was performed using a one-way ANOVA, followed by a least significant difference post hoc test (*n* = 3).

Cell apoptosis and caspase-3 activation have been implicated in the pathogenesis of AD. Upregulation of proapoptotic proteins and DNA fragmentation were also observed previously in the AD brain [58]. Accordingly, we examined the neuroprotective effect of QE on active caspase-3 expression in, and the apoptosis of, SH-SY5Y cells with H_2_O_2_-induced oxidative stress. Our results revealed that QE significantly reduced the expression of active caspase-3 in these cells (Figure 6A) in a dose–response trend as well as in reducing cell apoptosis (Figure 6B,C). At a concentration of 7.5 µM or above, the effect of QE did not show a statistical difference as the concentration went up. These results indicate that QE could inhibit the cell apoptosis due to the oxidative effect from H_2_O_2_.

## 4. Discussion

Under normal physiological conditions, an overexpression of free radicals disrupts the balance of ROS and antioxidants. Such an excess of free radicals can engender DNA damage, protein and lipid degradation, and neurodegenerative diseases, including AD, Parkinson’s disease, and amyotrophic lateral sclerosis. ROS are usually the source of oxidative stress in the body [59,60]. H_2_O_2_ is among ROS that reduce cell viability and increase the expression of apoptosis-related proteins such as active caspase-3 [61,62]. According to our preliminary experiment in which the cells were treated with 25, 30, 35, 40, and 45 μM H_2_O_2_. The results (figures not shown) showed that the survival rate of the cells were 69.3 ± 6.8%, 66.9 ± 2.3%, 67.1 ± 7.5%, 53.4 ± 5.2%, and 37.8 ± 1.7%. Additionally, the rate of cell apoptosis as shown in the expression of caspase-3 was elevated under 40 μM of H_2_O_2_. treatment. Therefore, we chose 40 μM of H_2_O_2_ as our treatment.

Oxidative stress reduces SIRT1 expression [63]. An increase in cellular ROS production results in a decrease in cellular mitochondrial biogenesis [27,64]. QE is a flavonoid [65] that could scavenge free radicals [66]. In our study, we found that SH-SY5Y cells exposed to H_2_O_2_ exhibited higher levels of ROS production than SH-SY5Y cells precultured with QE prior to H_2_O_2_ stimulation. Moreover, SH-SY5Y cells pretreated with an SIRT1 inhibitor and then treated with 10 μM QE exhibited reduced SIRT1, PGC-1α, and TFAM expression levels relative to SH-SY5Y cells treated with 10 μM QE followed by exposure to H_2_O_2_ stimulation. These findings demonstrate that QE pretreatment could reduce cellular ROS production and increase mitochondrial biogenesis by elevating the activity of the SIRT1-PGC-1α-TFAM pathway in SH-SY5Y cells exposed to H_2_O_2_.

Cellular mitochondria are produced within the cell body and supply energy through transportation in axons and dendrites [4]. Sheng indicated that the expression of mitochondrial biogenesis-related proteins such as PGC-1α and TFAM was lower in the postmortem brains of patients with AD than in the control group [27]. Additionally, studies have reported reduced mitochondrial biogenesis in the brains of APP transgenic mice [67,68]. These findings indicate that reduced mitochondrial biogenesis is a risk factor for neurodegenerative diseases.

Several studies have reported H_2_O_2_-induced cellular ROS overproduction in neuronal cells [27,69]. Antioxidants might increase mitochondrial biogenesis by inhibiting cellular ROS production. Mitochondria, which play a key role in energy homeostasis and ROS metabolism, require lifetime control and constant renewal [10]. The principal event of mitochondrial metabolism is the regulation of mtDNA transcription and translation, which is a complex coordinated process that involves at least two systems of transcription factors. PGC-1α and PGC-1β are the major regulatory proteins of this process in that they act as crucial factors linking several regulator cascades involved in the control of mitochondrial metabolism [70]. On the basis of the concept of antioxidant gene expression, PGC-1α is the major and only regulator of mitochondrial biogenesis.

PGC-1α regulates mitochondrial biogenesis by activating the expression of TFAM, which is a mitochondrial transcription factor. Once TFAM is activated, it induces mitochondrial biogenesis [27,71]. SIRT1 can execute deacetylation to induce PGC-1α activation by deaceylating acetyl groups [28]. We observed reduced PGC-1α expression after cell pretreatment with an SIRT1 inhibitor. This finding demonstrates a direct relationship between SIRT1 and PGC-1α [67,68].

Studies have reported that polyphenols can promote mitochondrial biogenesis by increasing the activity of SIRT1 agents such as resveratrol and epigallocatechin-3-gallate [72,73]. QE is polyphenolic flavonoid that can increase SIRT1 expression and activity by increasing nicotinamide phosphoribosyl transferase (NAMPT) activity and increasing NAD+ production [74]. A previous study fed mice with 12.5 or 25.0 mg/kg of QE for a week and then observed that SIRT1, PGC-1α, and mitochondrial biogenesis increased significantly in the brains of the mice [32]. Our results show that SIRT1, PGC-1α, and TFAM expression decreased, and that ROS production increased only in SH-SY5Y cells exposed to H_2_O_2_. In contrast, SIRT1, PGC-1α, and TFAM expression and mitochondrial biogenesis were increased significantly in SH-SY5Y cells that were precultured with QE at 2.5, 5.0, 7.5, and 10.0 μM and then exposed to H_2_O_2_. These results indicate that QE increases mitochondrial biogenesis by regulating the expression of mitochondrial-related proteins and reduces oxidative stress in SH-SY5Y cells exposed to H_2_O_2_.

Mitochondria are double-layer structures composed of inner and outer membranes [75]. ATP is primarily produced in electron transporter chains located in the inner membrane. Injecting 3-nitropropionic acid and 2-deoxy-D-glucose (2-DG) into the brains of transgenic mice with APP overexpression could inhibit the TCA cycle and glycolysis, which may induce increased BACE expression in the brains [29]. Our study revealed that SH-SY5Y cells treated with 40 μM H_2_O_2_ for 24 h exhibited not only a significant decrease in ATP and ROS production but also a significant increase in BACE expression compared with control cells. These results are consistent with those of a previous study [76]. In addition, Aβ production was regulated by BACE and ADAM10 [36]. Our findings indicate that ADAM10 expression decreased in SH-SY5Y cells exposed to H_2_O_2_ but that BACE and Aβ expression increased in these cells.

Previous studies have reported that ADAM10 is activated by a retinoic acid receptor (RAR); SIRT1 deacetylates RAR and subsequently increases ADAM10 expression [77,78]. In the present study, QE preculture may increase SIRT1 and ADAM10 expression in cells exposed to H_2_O_2_. A possible reason for this finding is that cellular PGC-1α expression inhibition could increase BACE mRNA production. Accordingly, these findings demonstrate that PGC-1α can regulate BACE transcription and translation [79]. In our study, cells precultured with QE exhibited increased PGC-1α expression and decreased BACE expression. This finding suggests that QE preculture could increase SIRT1 and PGC-1α expression to reduce BACE and could increase ADAM10 expression to reduce Aβ accumulation.

Aβ may engender SH-SY5Y cell apoptosis. Treating SH-SY5Y cells with 100 μM Aβ25–35 or 60 μM Aβ1–40 could induce cell dysfunction and a cell apoptosis cascade [80]. In our study, cells incubated with 40 μM H_2_O_2_ for 24 h exhibited a significant increase in active caspase-3 expression and apoptosis compared with those in the control group. However, preculturing the H_2_O_2_-exposed cells with QE ameliorated these results. This demonstrates that QE preculture could inhibit cellular apoptosis and reduce cell apoptosis cascades.

## 5. Conclusions

Our findings suggest that QE not only stimulated the expression of mitochondrial-related proteins in the H_2_O_2_-induced SH-SY5Y cells, such as SIRT1, PGC-1α, and TFAM but also activated mitochondrial biogenesis. Additionally, QE increased ADAM10 expression but reduced H_2_O_2_-induced reactive oxygen species production, apoptosis, β-site amyloid precursor protein cleaving enzyme 1 expression, and Aβ accumulation in the SH-SY5Y cells. Thus, the function of QE is closely related to elevating mitochondrial biogenesis-related proteins and reducing the damage caused by oxidative stress, making it a potential powerful option for protecting neuronal cells.

## Data Availability

The data are available upon request.
